# Editorial: The safety and efficacy of noninvasive brain stimulation in development and neurodevelopmental disorders

**DOI:** 10.3389/fnhum.2015.00544

**Published:** 2015-10-02

**Authors:** Lindsay M. Oberman, Peter G. Enticott

**Affiliations:** ^1^Neuroplasticity and Autism Spectrum Disorder Program, Department of Psychiatry and Human Behavior, E.P. Bradley Hospital and Warren Alpert Medical School, Brown UniversityProvidence, RI, USA; ^2^Cognitive Neuroscience Unit, School of Psychology, Deakin UniversityBurwood, VIC, Australia

**Keywords:** noninvasive brain stimulation, pediatric, transcranial direct current stimulation (tDCS), transcranial magnetic stimulation (TMS), safety

Noninvasive brain stimulation (NIBS) techniques including transcranial magnetic stimulation (TMS) and transcranial direct current stimulation (tDCS) are emerging as neuroscientific techniques that can be used as *in vivo* probes of brain function as well as therapeutic tools in a number of psychiatric and neurological disorders. Though much of the research and applications with these techniques have been applied to adult psychiatry and neurology, recent years have seen a number of researchers applying these tools to study brain development in typically developing children as well as those with neurodevelopmental and child psychiatric and neurological disorders. Clinical trials and case series designs have also been used to develop novel therapeutic interventions using these NIBS techniques in pediatric clinical populations and researchers are forming working groups dedicated to the application of NIBS to specific neurodevelopmental disorders (e.g., Autism Spectrum Disorder, Oberman et al., 2014a).

The papers in this research topic highlight the excitement in the field and the promise of these techniques both for the understanding of neurodevelopment (Pedapati et al., 2015) and neuropathology of neurodevelopmental disorders (Croarkin et al., 2014; Oberman et al., 2014b) as well as novel treatment development for neurodevelopmental disorders (Casanova et al., 2014; Gillick et al., 2014). This excitement and promise, however, is appropriately tempered by other papers in this research topic that highlight the unknown risks and potential ethical concerns related to applying these techniques in pediatric populations (Davis, 2014; Maslen et al., 2014).

A recent metaanalysis (Rajapakse and Kirton, 2013) reviewed the studies to date involving all rTMS protocols in children (approximately 1000 children have been studied across all rTMS protocols to date) and concluded “Its minimal risk, excellent tolerability and increasingly sophisticated ability to interrogate neurophysiology and plasticity make it an enviable technology for use in pediatric research with future extension into therapeutic trials.” This was supported by a paper in this topic highlighting the safety and tolerability of a specific paradigm, Theta Burst stimulation (Hong et al., 2015).

The most serious possible TMS-related adverse event is induction of a seizure. To date, 16 cases of TMS-induced seizures have been reported out of tens of thousands of examined subjects over the past 25 years. Overall the risk of seizure is considered to be less than 0.01% across all patients and all paradigms (Rossi et al., 2009). The risk of overall adverse event burden from TMS, however, may be underestimated due to the lack of systematic identification, tracking, and reporting of adverse events in study publications. Thus, the safety, tolerability, and efficacy have not been characterized sufficiently to justify off-label clinical use of NIBS, especially in pediatric populations. At this point, use of these technologies either for investigational or clinical use should be under the context of an investigational device exemption (IDE) or IRB approved research trial. Unfortunately, there have been instances of “do-it-yourself” brain stimulation devices entering the marketplace, raising the possibility that these techniques will be applied to individuals with neurodevelopmental disorders without an evidence-base, regulatory oversight, or appropriate expertise.

Despite the therapeutic promise of repetitive TMS for neurodevelopmental disorders, translation to “treatment-based” protocols poses a number of important challenges and complexities. For instance, there are various considerations in selecting pulse sequences (e.g., frequency, intensity), regions of stimulation, and coil type, each combination of which is likely to have different efficacy and side-effect profiles. While TMS has been the primary technique employed in neurodevelopment thus far, electrical stimulation techniques (e.g., tDCS, transcranial alternating current stimulation [tACS]) have very different mechanisms of action and risk profiles (e.g., seizure induction is not generally indicated in tDCS/tACS). Brain stimulation protocols can also have differing effects across participants, and these effects might be exacerbated when considering the heterogeneity of neurodevelopmental disorders such as autism spectrum disorder (ASD).

Another important factor to consider in trialing therapeutic interventions is the optimal age of intervention. It might be argued that the greatest effects will be seen if NIBS is applied early in development, when the brain is considered more plastic. As noted, however, there are important ethical and feasibility concerns around NIBS in children. At present, a relatively small number of typically developing children and children with neurodevelopmental disorder have undergone NIBS. Single pulse TMS has been applied to study development of corticospinal projections in neonates within hours of birth (Eyre et al., 2001), however, repetitive (rTMS) has been limited to older children and adolescents. Thus, any interaction between repetitive brain stimulation and neurodevelopment is currently unknown. This is particularly important in the context of developmental disorders where in most cases the developmental neuropathology has yet to be fully elucidated.

In conclusion, there is an obvious need for further research in this area. Specifically, studies focusing on developmental trajectories and how the effects of NIBS change across childhood would be extremely useful. The use of NIBS in children is a burgeoning field whose full potential has yet to be realized. The papers in this research topic speak to both the promise and the challenges that researchers and clinicians face when applying NIBS techniques to study typical development, developmental pathophysiology, and as potential nonpharmacological, brain-based treatments for neurodevelopmental disorders.

## Author contributions

LO and PE co-wrote the manuscript.

### Conflict of interest statement

The authors declare that the research was conducted in the absence of any commercial or financial relationships that could be construed as a potential conflict of interest.

## References

[B1] Casanova M. F. Hensley M. K. Sokhadze E. M. El-Baz A. S. Wang Y. Li X.. (2014). Effects of weekly low-frequency rTMS on autonomic measures in children with autism spectrum disorder. Front. Hum. Neurosci. 8:851. 10.3389/fnhum.2014.00851 25374530 PMC4204613

[B2] Croarkin P. E. Nakonezny P. A. Lewis C. P. Zaccariello M. J. Huxsahl J. E. Husain M. M.. (2014). Developmental aspects of cortical excitability and inhibition in depressed and healthy youth: an exploratory study. Front. Hum. Neurosci. 8:669. 10.3389/fnhum.2014.00669 25228870 PMC4151107

[B3] Davis N. J. (2014). Transcranial stimulation of the developing brain: a plea for extreme caution. Front. Hum. Neurosci. 8:600. 10.3389/fnhum.2014.00600 25140146 PMC4122183

[B4] Eyre J. A. Taylor J. P. Villagra F. Smith M. Miller S. (2001). Evidence of activity-dependent withdrawal of corticospinal projections during human development. Neurology 57, 1543–1554. 10.1212/WNL.57.9.1543 11706088

[B5] Gillick B. T. Kirton A. Carmel J. B. Minhas P. Bikson M. (2014). Pediatric stroke and transcranial direct current stimulation: methods for rational individualized dose optimization. Front. Hum. Neurosci. 8:739. 10.3389/fnhum.2014.00739 25285077 PMC4168687

[B6] Hong Y. H. Wu S. W. Pedapati E. V. Horn P. S. Huddleston D. A. Laue C. S.. (2015). Safety and tolerability of theta burst stimulation vs. single and paired pulse transcranial magnetic stimulation: a comparative study of 165 pediatric subjects. Front. Hum. Neurosci. 9:29. 10.3389/fnhum.2015.00029 25698958 PMC4316715

[B7] Maslen H. Earp B. D. Cohen Kadosh R. Savulescu J. (2014). Brain stimulation for treatment and enhancement in children: an ethical analysis. Front. Hum. Neurosci. 8:953. 10.3389/fnhum.2014.00953 25566011 PMC4270184

[B8] Oberman L. M. Enticott P. G. Casanova M. F. Rotenberg A. Pascual-Leone A. McCracken J. T. (2014a). Transcranial magnetic stimulation (TMS) therapy for autism: an international consensus conference held in conjunction with the international meeting for autism research on May 13th and 14th, 2014. Front. Hum. Neurosci. 8:1034. 10.3389/fnhum.2014.01034 25642178 PMC4295436

[B9] Oberman L. M. Pascual-Leone A. Rotenberg A. (2014b). Modulation of corticospinal excitability by transcranial magnetic stimulation in children and adolescents with autism spectrum disorder. Front. Hum. Neurosci. 8:627. 10.3389/fnhum.2014.00627 25165441 PMC4131188

[B10] Pedapati E. V. Gilbert D. L. Horn P. S. Huddleston D. A. Laue C. S. Shahana N.. (2015). Effect of 30 Hz theta burst transcranial magnetic stimulation on the primary motor cortex in children and adolescents. Front. Hum. Neurosci. 9:91. 10.3389/fnhum.2015.00091 25762919 PMC4340218

[B11] Rajapakse T. Kirton A. (2013). Non-invasive brain stimulation in children: applications and future directions. Transl. Neurosci. 4, 217–223. 10.2478/s13380-013-0116-3 24163755 PMC3807696

[B12] Rossi S. Hallett M. Rossini P. M. Pascual-Leone A. Safety of TMS Consensus Group. (2009). Safety, ethical considerations, and application guidelines for the use of transcranial magnetic stimulation in clinical practice and research. Clin. Neurophysiol. 120, 2008–2039. 10.1016/j.clinph.2009.08.016 19833552 PMC3260536

